# Supplementary material for: Perinatal anxiety and compromised bond: A qualitative study of cultural scripts, structural barriers and maternal emotional negotiations in Pakistan
Source: Glob Ment Health (Camb). 2026 Feb 11;13:e44. doi: 10.1017/gmh.2026.10152 (PMC12973247; doi:10.1017/gmh.2026.10152)
Supplement: Liaqat et al. supplementary material [file S2054425126101526sup001.docx]

**FINAL INTERVIEW GUIDE (ENGLISH VERSION) Supplementary Material**

**Perinatal Anxiety and Maternal Bonding Study**

**Interviewer Note:** At the start, assign a unique participant ID (e.g., IDI-01). Use this ID on all recordings, transcripts, and notes. Begin with the Opening Script and consent process as in your original guide.

## ****SECTION 1: INTRODUCTION AND CONSENT****

1. Introduce yourself and explain the purpose of the interview.
2. Ensure confidentiality and obtain informed consent.
3. Explain the structure of the interview and the estimated time.
4. Encourage openness and honesty, ensuring there are no right or wrong answers.

**Opening Script:**
“I am interested in understanding your emotional and caregiving experiences during pregnancy or after birth. There are no right or wrong answers.”

## ****SECTION 2: BACKGROUND INFORMATION****

1. To begin, could you tell me a little about yourself?
   (Probe: age, family structure, who lives at home, education, work status)
2. Can you describe your current pregnancy?
   (Probe: weeks, planned/unplanned, any complications)
3. What was your initial reaction and feeling when you found out you were pregnant?
4. How did your partner and immediate family react to the news?

## ****SECTION 3: EMOTIONAL EXPERIENCES DURING PREGNANCY AND POSTPARTUM****

3.1 How would you describe your overall emotional state during this pregnancy/postpartum period?
3.2 What kinds of worries, fears, or stresses have been most present for you?
3.3 Have there been times when you felt overwhelmed, tearful, or emotionally low?
  Can you describe what that feels like and what triggers it?
3.4 What helps you cope or feel better when you have these difficult emotions?

**Interviewer Note:** Pause if needed; offer support; respect if they decline to answer.

## ****SECTION 4: CAREGIVING EXPERIENCE****

4.1 Walk me through a typical day for you now. What is it like?
4.2 How has your physical recovery and tiredness affected your daily life and your feelings?
4.3 Do you feel you have had enough time and space to rest, to heal, and to just be with your baby?
4.4 (If employed) How has the transition back to work (or thinking about it) been for you emotionally?

## ****SECTION 5: FAMILY AND SOCIAL CONTEXT****

5.1 How would you describe the support you receive from your husband?
  (Probe: emotional sharing, practical help with chores/baby)
5.2 How involved are your in-laws or joint family in your daily life and caregiving?
  What has that experience been like for you?
5.3 In decisions about the baby or your own rest, how much do you feel you can do what you think is best versus what others expect?
5.4 Has the baby’s gender been a topic of discussion in your family or community?
  Can you tell me about that?

## ****SECTION 6: CULTURAL CONTEXT & BONDING****

6.1 What does being a “good mother” mean in your family or community?
  (Have you felt pressure to meet certain expectations?)
6.2 Did you follow any specific postpartum customs?
  What was that experience like for you (helpful, restrictive, or both)?
6.3 How would you describe your sense of connection or closeness with your baby now?
6.4 Is the bond with your baby a constant feeling, or does it change?
6.5 Are there moments you feel more or less connected?
6.6 Has financial pressure or access to healthcare been a source of stress for you?

## ****SECTION 7: SUPPORT & RECOMMENDATIONS****

7.1 Looking beyond family, who or what has been your most important source of support?
7.2 Have you shared your emotional ups and downs with a healthcare provider (doctor, nurse, midwife)?
  What was their response?
7.3 **(Ask if possible)**
  in any of your relationships, do you ever feel afraid, controlled, or unsafe?
  Has anyone threatened or hurt you?
7.4 Based on all we’ve talked about, what is the one thing you wish your family understood better about what you’re going through?

## ****SECTION 8: SUPPORT SYSTEMS AND HELP-SEEKING****

8.1 Who has been most supportive for you emotionally or practically?
8.2 How have healthcare workers responded to your emotional concerns?

## ****SECTION 9: CLOSING QUESTIONS****

9.1 What should families or healthcare providers do differently to support mothers?
9.2 Is there anything else you would like to share about your emotional experience?
9.3 Reflecting on our conversation, is there any important part of your experience we haven’t touched on?
9.4 How has it felt for you to talk about these things today?
9.5 Do you have any questions for me?

## Interviewer Note: Thank you very much for sharing your experiences with me today. I truly appreciate your time,and trust. Your insights are valuable and will help us better understand and support mothers’ emotional wellbeing. If talking about these topics brought up any distress, please let me know so I can connect you with appropriate support

**Supplementary Table 1. Conceptual Mapping of Qualitative Indicators of Bonding Difficulty to the Postpartum Bonding Questionnaire (PBQ) Framework**

| Qualitative Indicator (Thematic Code) | Illustrative Quote (Participant ID) | Mapped PBQ Domain / Subscale (Brockington et al., 2001) | Rationale for Mapping & Coding Note |
| --- | --- | --- | --- |
| Persistent emotional blankness / lack of affection | *"I just felt blank… I didn't feel like a mother."* (IDI-07) | Factor 1: Impaired Bonding (e.g., "I feel close to my baby") | Coded when narratives described a pervasive absence of expected positive emotion or connection toward the infant. |
| Irritability / anger during caregiving | *"I became very irritated… even felt angry toward my baby."* (IDI-17) | Factor 2: Rejection and Anger (e.g., "I feel angry with my baby") | Coded when frustration or anger was directed at the infant or the caregiving role, indicating a strained relationship. |
| Infant-focused anxiety / hypervigilance | *"I never left my baby alone, not even for a minute. I had this fear..."* (IDI-02) | Factor 4: Infant-Focused Anxiety (e.g., "I am afraid of my baby") | Coded for excessive worry, checking, or protective behaviors stemming from anxiety rather than affectionate attunement. |
| Avoidance of physical closeness | *"I didn’t feel any connection. I couldn’t even look at my baby..."* (IDI-07) | Factor 1: Impaired Bonding (e.g., items reflecting emotional/physical distance) | Coded for narratives describing active withdrawal from holding, eye contact, or other forms of physical proximity. |
| Guilt about maternal feelings | *"I felt guilty for not feeling the happiness I thought I should..."* (IDI-12) | Factor 3: Anxiety About Care / General PBQ Guilt Items | Coded when distress was focused on the perceived inadequacy of one's own maternal feelings or actions. |
